# Ultrasensitive signal detection by a guanylyl cyclase chemoreceptor

**DOI:** 10.1186/2050-6511-16-S1-A20

**Published:** 2015-09-02

**Authors:** U Benjamin Kaupp

**Affiliations:** 1 Department of Molecular Sensory Systems, Center of Advanced European Studies and Research (caesar), Bonn, Germany

## Background

Sperm navigate to the egg in a gradient of a chemoattractant for fertilization – a mechanism called chemotaxis. In sea urchin, the chemoattractant peptide binds to a chemoreceptor guanylyl cyclase (GC) on the sperm surface. Activation of the GC initiates a sequence of signaling events that eventually results in Ca2+ influx and a change in swimming direction. We studied the GC properties that allow sperm to track the chemoattractant with single-molecule precision on a millisecond time scale. A high density (9•103 GC molecules/µm2) and a subnanomolar ligand affinity provide a high ligand-capture efficacy. The sperm surface represents an almost perfect absorber. The peptide-induced GC activity is terminated by multiple dephosphorylation steps, which provide a means of precise lifetime control and, thereby, reduces “molecular noise”. Several experiments suggest that GC undergoes auto-dephosphorylation. Future experiments need to clarify, whether the GC entertains phosphatase and kinase activity, possibly in the kinase-homology domain (KHD). The turnover of cGMP synthesis of 72 cGMP molecules/sec or about 11 cGMP molecules/GC*/lifetime is sufficient to open a few cGMP-gated channels and to produce a unitary voltage response of about 2 mV. The receptor GC can bind the ligand over six orders of magnitude of concentrations. The shallow binding curve might reflect negative cooperativity among binding sites; alternatively receptor population might be composed of a mixture of receptors with a range of K_1/2_ values.

**Figure 1:**
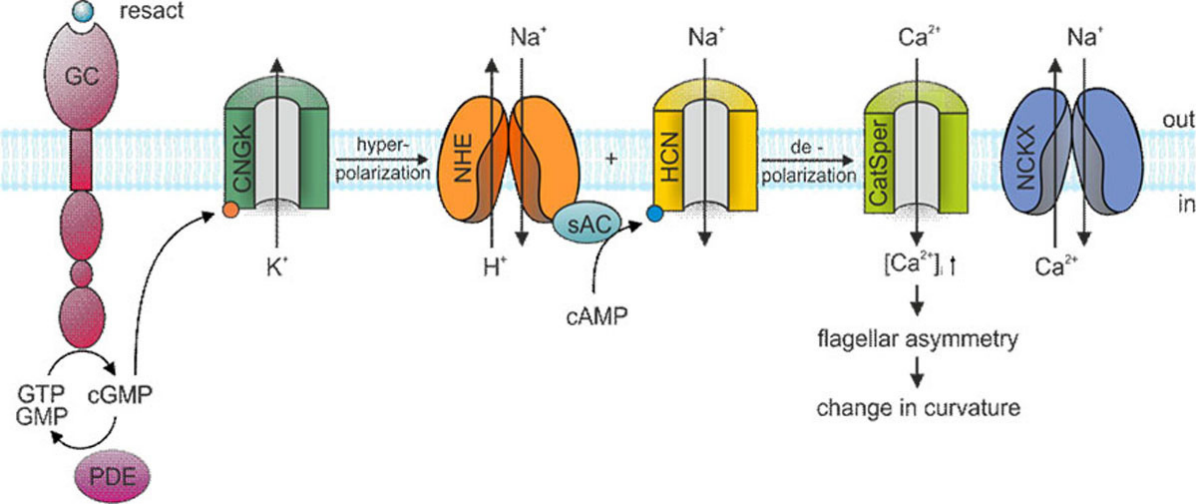
Cellular signaling during chemotaxis in sea urchin sperm. The signaling events are initiated by binding of the ligand to the receptor GC. The rise of cGMP opens K^+^-selective cyclic nucleotide-gated channels (CNGK). The ensuing hyperpolarization activates a voltage-dependent Na^+^/H^+^ exchanger (NHE) and a pacemaker channel (HCN). The resulting alkalization by NHE and depolarization by HCN opens a sperm-specific Ca^2+^ channel (CatSper). Ca^2+^ is exported by a Na^+^/Ca^2+^-K^+^ exchanger (NCKX).

